# Epigenetic Regulation of Root-Associated Microbiota: Mechanisms and Horticultural Applications

**DOI:** 10.3390/plants15060938

**Published:** 2026-03-19

**Authors:** Subo Tian, Ning Zhang, Guiyu Lin, Xiaoli Cheng, Fubin Wang, Peipei Chang, Golam Jalal Ahammed, Qinghua Shi, Wen-Feng Nie, Yan Zhang

**Affiliations:** 1 College of Horticulture Science and Engineering, Shandong Agricultural University, Tai’an 271018, China; 2 Jia Sixie College of Agriculture, Weifang University of Science and Technology, Weifang 262700, China; 3 Jining Academy of Agricultural Sciences, Jining 272031, China; 4 Dezhou Academy of Agricultural Sciences, Dezhou 253055, China; 5 College of Horticulture and Plant Protection, Henan University of Science and Technology, Luoyang 471023, China

**Keywords:** epigenetic, horticulture, DNA methylation, Microbiome

## Abstract

The dynamic interaction between plants and their root-associated microbiota represents a sophisticated and profound biological communication that regulates plant development and the formation of adaptation to the surrounding environment. These interactions function as critical regulators of multiple physiological processes, finally influencing soil fertility and agricultural productivity. Plants have evolved epigenetic networks that regulate beneficial plant–microbe interactions through regulating immune responses, gene regulation, and metabolite production to enhance stress tolerance and soil adaptation. These regulations collectively govern microbial colonization patterns while establishing reciprocal feedback loops through root exudate–microbe interactions. This review systematically updates contemporary advances in understanding how epigenetic modifications shape rhizosphere microbiome composition and function, and discusses their potential applications in enhancing the yield and quality of horticultural crops, as well as in mitigating continuous cropping obstacles.

## 1. Definition of Root-Associated Microbiome in Plants

Root-associated microbiota enhance plant productivity through nitrogen fixation, nutrient solubilization, and stress resilience. Current applications utilize plant growth-promoting rhizobacteria (PGPRs) and mycorrhizal fungi to improve yields and disease resistance, showing particular promise in addressing continuous cropping obstacles—a critical challenge in intensive horticulture. By modulating microbial communities, beneficial microbes can suppress pathogens, degrade toxins, and restore degraded soils. Emerging approaches combine microbial consortia with organic amendments to rehabilitate rhizosphere ecosystems.

Plants can be colonized by diverse microorganisms, resulting in beneficial, detrimental, or neutral effects on the host [1,2]. Microbiota identified from plants are diverse, such as bacteria [2], fungi [3], oomycetes [4,5], and viruses [1]. A variety of microbial species associated with the shoot system are known as the phyllosphere microbiome, including colonizing epiphytes and endophytes [6]. Among them, the phyllosphere microbial community needs to deal with natural stimuli such as ultraviolet (UV) radiation, light intensity, temperature changes, water fluctuation, nutrient availability, microbial interactions [7], and human actions, such as pesticide spraying and other aspects of agricultural management [6,8,9]. These activities also have a great impact on plant root-related microbiota and the soil environment. In turn, long-term imbalanced fertilization maintains stable soybean yields by regulating the dynamic assembly of rhizosphere microbiota (particularly plant growth-promoting microbial clusters enriched under low-nitrogen conditions), providing quantitative evidence for microbiome management in sustainable agriculture [10].

### 1.1. The Composition and Regulation of Root-Associated Microbiome

Plant-associated soil microbial communities are closely associated with the roots, and exhibit a spatially organized architecture, comprising three distinct ecological niches: rhizosphere microbiome inhabiting the soil–root interface, epiphyte microbiota colonizing the root surface/rhizoplane, and endophyte microbiota residing within the root interior/endosphere [9,11,12], which are generally designated as the root-associated microbiome. A three-step enrichment model elucidating the assembly mechanisms of plant root-associated microbiomes was proposed, including the microbial enrichment of roots to form a rhizosphere microbiome, the selection of a microbe–host interaction by the plant host to assemble the rhizoplane microbiome, and the delivery of microorganisms into the root interior to establish an endosphere microbiome [12,13]. The assembly processes of the root-associated microbiome are modulated by biotic stresses [13], environmental conditions [14], bacterial communities [15], and plant genomic background [2,16,17,18,19].

### 1.2. Biological Functions of Root-Associated Microbiome

Healthy plant roots are normally colonized by a diverse and abundant microbial community that can, in turn, regulate a variety of physiological activities in host plants. Rhizosphere microbial communities enhance plant fitness through a variety of mechanisms, including phytohormone-mediated growth promotion, biodegradation of environmental pollutants, modification of plant immune systems, and improvements in abiotic and biological stress resistance [9]. The root microbiota regulates rice tillering by producing cyclic dipeptides that activate the strigolactone signaling pathway, revealing a novel mechanism by which the microbiome modulates key agronomic traits in crops [20]. In addition, nutrient uptake by roots is related to the rhizosphere microbiome [21,22]. The microbiome can alter phenotypes of host plants and expand their genomic and metabolic capabilities [8]. Recently, several studies demonstrated that the rhizosphere microbial communities possess a strong functional potential to extend plant phenotypes [8,23], suggesting that microbial communities are increasingly becoming important regulatory factors for plant adaptation to environmental challenges. Moreover, the underground portion of the plant is embedded in and physically integrated into the soil, and is influenced by edaphic properties [1], environmental conditions (i.e., climate factors), diseases, insect pests, plant species, plant genotypes, and root phenotypes [9]. The plant–soil system affects microbial diversity and population, thereby regulating the functions and services of a terrestrial ecosystem, such as biogeochemical cycling, primary production and plant fitness [24]. In addition, root-associated microbiomes could be used to improve soil aggregation in management practices [25]. Moreover, plant-released volatile organic compounds (VOCs) function as important signaling molecules that regulate ecological interactions among the root-associated microbiome [26]. Therefore, understanding the biological functions and regulation of root-associated microbiome will provide important guidance for expanding plant plasticity and soil stabilization in management practices [8], which exerts significant impacts on global food supply and security [27].

## 2. Epigenetic Modifications Regulate Root-Associated Microbiota in Plants

### 2.1. The Major Epigenetic Modifications in Plants

Epigenetic modifications, such as DNA methylation, have been identified as important regulators that promote plant growth [28].

In plants, DNA methylation predominantly occurs in three sequence contexts: CG, CHG, and CHH contexts (where H is A, C, or T). De novo DNA methylation is primarily achieved through the RNA-directed DNA methylation (RdDM) pathway [29]. In plants, the maintenance of CG, CHG, and CHH methylation is regulated by METHYLTRANSFERASE 1 (MET1), and CHROMOMETHYLASEs CMT3 and CMT2, respectively [30,31]. DOMAINS REARRANGED METHYLTRANSFERASE DRM2 is responsible for de novo DNA methylation at all three cytosine sequence contexts [32]. On the contrary, methylated cytosines could be removed from DNA sequences by 5-methylcytosine DNA glycosylases, such as REPRESSOR OF SILENCING 1 (ROS1) [33], DEMETER (DME) [34], and DEMETER-LIKE proteins (DML2 and DML3) [35,36]. In eukaryotes, genomic DNA is structurally organized in the form of nucleosomes. Each nucleosomal core particle contains ~147 bp of DNA wrapped around an evolutionarily conserved histone octamer comprising two copies each of H2A, H2B, H3, and H4 [37]. Multiple covalent alterations of histone proteins regulate transcriptional activity, such as histone methylation, histone acetylation, and histone variants (e.g., H2A.Z and H3.1) [31,38]. These epigenetic modifications function as critical molecular switches governing developmental programs in plants, primarily through two mechanistic routes: (i) modulating histone–DNA binding affinity, and (ii) creating docking sites for regulatory protein complexes [39,40,41]. Modifications of individual histones may alter nucleosome structure and affect chromatin environment, thereby affecting gene expression. This modification has been functionally linked to diverse physiological phenomena, such as fruit ripening [42,43,44], fruit expansion [45], heat stress [46], and autophagy [47]. The histone protein repertoire extends beyond standard isoforms, with specialized H3 and H2A variants being ubiquitously present across eukaryotic species. These variant proteins confer structural plasticity to chromatin architecture, enabling stage-specific functions during cell cycle progression and participation in essential biological pathways [48,49,50].

Non-coding RNAs (ncRNAs) are transcribed from genomes and play crucial regulatory roles in chromatin organization and genetic information flow [51]. Distinct from protein-coding mRNAs, ncRNAs lack translational capacity but exhibit diverse functional mechanisms. According to their expression patterns and biological roles, ncRNAs are broadly classified into two functional categories: (i) constitutively expressed housekeeping ncRNAs, including rRNAs, tRNAs, small nucleolar RNAs (snoRNAs), and small nuclear RNAs (snRNAs), which maintain fundamental cellular activities; and (ii) conditionally expressed regulatory ncRNAs that respond to developmental cues or environmental stimuli [52]. The regulatory ncRNAs are further categorized by size into small ncRNAs (<200 bp) and long ncRNAs (lncRNAs; >200 bp), each with distinct modes of action [53].

In eukaryotes, chromatin remodeling complexes dynamically modulate chromatin architecture to regulate DNA accessibility and precise gene expression. In plants, chromatin remodelers can be broadly classified into four major groups: (i) the SWR1 complex, which mediates histone variant exchange (e.g., H2A.Z deposition) [54,55]; (ii) the INO80 complex involved in nucleosome sliding and eviction [56,57]; (iii) the NuA4 histone acetyltransferase complex, which epigenetically modifies histones to promote transcriptional activation [58]; and (iv) the SWI/SNF (switch/sucrose non-fermentable) complex, a well-characterized ATP-dependent remodeler that repositions nucleosomes [59]. These complexes utilize energy derived from ATP hydrolysis and histone post-translational modifications (PTMs) to restructure nucleosome–DNA interactions [60,61], thereby fine-tuning transcription factor binding and activity. Moreover, these remodelers serve as critical regulators of transcriptional reprogramming in response to phytohormone signaling and environmental challenges [62]. *Trichoderma harzianum* T22 triggers transcriptomic and epigenetic reprogramming in tomato roots, enhancing growth and stress tolerance [63].

Currently, recent studies have found that epigenetic modifications that interact with root-associated microbiota in plants are mainly achieved through DNA methylation, histone modification, histone variation, non-coding RNA, and chromatin remodeling complexes (Table 1). These diverse epigenetic modifications coordinately modulate the chromatin landscape to control gene expression.

### 2.2. The Roles of the Interaction Between Microbial Community and Epigenetic Modifications in Plants

The diverse gene pool of plant-associated microorganisms interacts with the host genome to induce epigenetic effects, ultimately leading to the establishment of adaptive phenotypes [67]. It is increasingly recognized that epigenetic phenotypic plasticity plays a pivotal role in plant evolution, hinting that the application of epigenetic modification may enrich the diversity of microbial-community-mediated plant growth and adaptation in production practice. Horticultural products, including vegetables and fruits, provide essential nutrients and energy for humans [68,69]. The market monitoring of horticultural products, including quality index, yield index, flavor index and storage index, as well as the development of the world economy and the improvement of living standards, all promote people to pursue more abundant product characteristic plasticity. The oomycete pathogen *Phytophthora sojae* employs PsDMAP1-/PsTIP60-mediated H4K16 acetylation to modulate reactive oxygen species (ROS) signaling pathways, thereby achieving virulence adaptation to host plants [70]. Through stimulating rhizosphere sulfur respiration to produce H2S, sodium butyrate epigenetically enhances rice drought tolerance via histone acetylation regulation [66]. Both epigenetic markers and the microbiome are key regulatory mediators of plant phenotypic development and establishment [8,23,71]. Although epigenetic modifications involving the regulation of vegetable products are being studied [72], the contribution of root-associated microbiomes to product characteristics and plasticity remains unclear. In addition, epigenetic modifications can induce phenotypic plasticity of horticultural plant hosts [2], and the improved host plants can further promote the expansion of horticultural trait plasticity and the improvement of quality through the interaction with microorganisms (Wilkinson and Ton, 2020) [73]. These factors highlight the interest in studying and explaining microbial–epigenetic factor interactions and plant–microbial interactions in plants with a view to facilitating future epigenetic breeding.

## 3. Interplay Between Epigenetic Modifications and the Root-Associated Microbiome

**Figure 1:**
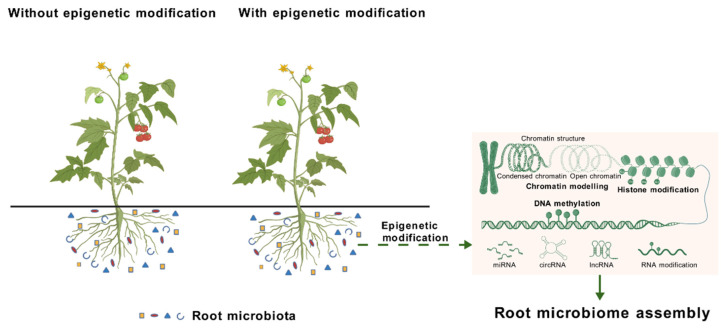
An overview of the interactions between plant epigenetic modifications and root microbiome.

Epigenetic regulation orchestrates diverse biological processes in plants, including vegetative and reproductive growth, seed maturation, flowering, fruit ripening, carotenoid metabolism, and response to abiotic and biotic stress [31,74,75,76]. However, due to the inherent complexity of biological systems and the dynamic nature of epigenetic modifications, the potential effects of epigenetics on microbial community formation and their regulatory mechanisms remain underestimated. Until recently, epigenetic markers (i.e., active DNA demethylation, Dicer-like (DCL) proteins in the RdDM pathway, and histone demethylase IBM1) have been found to regulate the metabolism of root exudates and composition of the root-associated microbiome in *Arabidopsis* and/or tomato [2,16,77], which provides new clues to the regulatory mechanism of epigenetic modifications on root-associated microbiomes (Figure 1). Long-term epigenetic reprogramming in plant–microbe interactions: Plant growth-promoting bacteria (PGPBs) induce persistent DNA methylation changes that mediate growth enhancement processes [65].

### 3.1. The Biological Function of Epigenetic Manipulation in Plant Immunity Against Phytopathogens

The innate immune system in plants plays a crucial role in responding to multiple pathogenic infections, facilitating adaptation to adverse conditions and environmental challenges by coordinating the balance between plant development and defense processes. The plant immune system involves many regulatory factors, such as signal transduction modules, transcriptional networks, and hormone interactions. In general, plants have evolved a two-layered surveillance system to defend against pathogens. Microbial signals are recognized by two types of receptors to activate the plant immune system. The first type is the conserved microbe-/pathogen-associated molecular patterns (PAMPs), which recognize microbial signals and activate PAMP-triggered immunity (PTI). The second type includes intracellular nucleotide-binding leucine-rich repeat (NLR) receptors, which detect pathogen effector proteins and activate a more robust defense mechanism termed effector-triggered immunity (ETI) [78]. The corresponding pattern of signal perception activation is somewhat different; however, recent studies have confirmed that PTI and ETI cross-talk by sharing multiple elicitors, creating a synergistic amplification loop that enhances the plant’s defensive capacity against microbial pathogens [79,80,81,82], further indicating that PTI and ETI are necessary for plants to withstand biotic and abiotic challenges.

The pivotal role of epigenetic regulation in modulating plant immunity has been well established, with DNA (de)methylation, histone modifications, and chromatin remodeling emerging as key players in fine-tuning immune responses through different mechanisms. DNA methylation and active DNA demethylation collectively regulate plant immune responses through modulating the transcription of immune-related genes mediated by DNA methylation under biological stresses [83,84,85,86]. This regulation occurs through two primary mechanisms: (i) modulation of transposable element (TE)/repeat sequence methylation in promoter regions, and (ii) control of intronic TE methylation within gene bodies [87,88]. For histone modification, it was reported to respond to virulent bacterial pathogens, *Pst* DC3000 and *Xanthomonas oryzae pv. Oryzae* [89,90]. Moreover, H3K9me2 interacts with DNA methylation to produce full-length and short-length *RPP7* transcripts that can regulate plant immunity [91], indicating that DNA and histone methylation coregulate the expression of plant immune-responsive genes through their synergistic interplay. The ncRNAs, such as small interfering RNAs (siRNAs), microRNAs (miRNAs), and lncRNAs, are also reported to regulate plant immunity [92,93]. For example, in tomato, overexpression of lncRNA16397 confers enhanced resistance against *Phytophthora infestans* by mitigating oxidative stress and preserving membrane integrity [93]. Moreover, mounting evidence proves the crucial involvement of chromatin remodeling processes in modulating plant immune responses. Specifically, SWI2/SNF2-Related 1 chromatin remodeling complex (SWR1-C) mediates the deposition of H2A.Z histone variants to specific chromosomal regions, primarily through ATP-dependent H2A-H2A.Z exchange [94]. The depletion of H2A.Z, as well as dysfunction of SWR1 COMPLEX 6 (SWC6) and Photoperiod-Independent Early Flowering1 (PIE1), can reduce basal resistance in *Arabidopsis* [95]. It is worth noting that although ncRNAs and chromatin remodeling complexes clearly participate in biological stress adaptation and plant immune regulation, the underlying mechanisms are poorly understood.

Substantial research advances in recent decades have elucidated the sophisticated regulatory networks governing plant immune responses. Previous studies have shown that plants usually rely on the immune response system to resist damage caused by pathogens through absorbing beneficial rhizosphere microorganisms [96]. The application of *Pst* DC3000 could partially regulate the metabolites in the exudates of roots [97]. The colonization of specific beneficial microbes in the root system can reduce the susceptibility of natural plants to pathogens or insect herbivores through this induced resistance, thus ensuring the healthy growth of plants [98]. The epigenetic factor Increase Bonsai Methylation 1 (IBM1), a demethylase of H3K9 methylation marker, can reduce the abundance of *Pseudomonas* microbe members and reshape the root microbiota [77], indicating the bio-function of epigenetic modifications in plants and microbiome. Therefore, one may speculate that the epigenetic regulation can effectively mediate the composition of plant-associated microbiomes, and the change in microbial population, in turn, influences the physiological characteristics of plants and their adaptability to environmental conditions, which supports the significant contribution of epigenetic regulation to the plant–microbiome crosstalk in soils. However, in contrast to extensively studied effects of epigenetic modifications on plant immunity against pathogens, the roles of epigenetic modifications in reshaping the root microbiome are still unclear.

### 3.2. Epigenetic Manipulation of Plant Metabolism Rearranges the Root-Associated Microbiome Landscape

Root exudates and their interactions with microbiota are critical to plant growth and development. Root exudates contain a variety of organic compounds that create a nutrient-rich environment for rhizosphere soil bacteria [99]. Root exudates refer to diffused compounds passively exported by roots [100], which contain a range of primary and secondary metabolites and are an important driving force of rhizosphere microbial community assembly [9]. Root exudates also serve as indirect indicators of both photosynthetic carbon fixation efficiency and total nitrogen utilization [9,100,101]. Emerging evidence reveals that host DNA methylation/demethylation partially governs the assembly of rhizosphere-associated bacterial communities. Genetic disruption of the DNA demethylases (e.g., ROS1, DML2, and DML3) impairs plant responsiveness to *Bacillus megaterium* strain YC4, abolishing its growth-promoting effects [2]. Intriguingly, RdDM-deficient mutants (*nrpd1-3* and *nrpe1-11*) fail to alter YC4 activity and maintains full responsiveness to YC4-mediated growth promotion [2], revealing that DNA methylation-regulated beneficial microbiota assembly is a sophisticated process that may exhibit partial dependence on root metabolite profiles and/or bacterial taxa specificity.

Plant metabolism depends on the multicellularity of metabolism, the subcellular compartmentation of metabolic pathways, and the redundancy of pathway functions [102]. Investigating the crucial regulatory factors of root metabolites offers a rational scientific foundation for epigenetic modification, thereby facilitating beneficial microbiota recruitment and optimizing microbial utilization of plant metabolites. Emerging evidence indicates that root exudates can inversely modulate gene expression [103] and epigenetic factors [2,16,77] in host plants. DNA methylation dynamics influence secondary metabolite biosynthesis and alter inositol accumulation, a key carbocyclic sugar involved in cellular signaling [2]. It was shown that the plant–rhizobacteria interaction is disturbed by environmental conditions, and the reduction in plant–rhizobacteria interactions may further weaken the response to heat stress by affecting plant growth [104]. Thus, the synergistic action of primary and secondary metabolites may shape an optimal rhizosphere microenvironment for extensive microbial colonization and reciprocal root–microbiome crosstalk. These metabolites serve as pivotal molecular bridges, integrating epigenetic regulation with microbial community assembly to establish self-reinforcing feedback loops essential for plant–microbiome homeostasis.

## 4. Significance of Epigenetic Modification in the Regulation of Root-Associated Microbiome and Soil Environment in Cropping Systems

The agronomic traits of horticultural crops (e.g., vegetables and fruits) are closely correlated with soil biodiversity, which is mediated by the structural and functional dynamics of root-associated microbial communities [105]. In addition, the formation of horticultural product quality is also strictly controlled by genetic and/or epigenetic inheritance and evolution. This indicates that combining artificial control of soil microorganisms with genetic approaches such as gene editing and epigenetic modification is a potentially feasible, practical strategy to improve the traits and quality of horticultural products.

### 4.1. Soil Microbiological Effects in the Management Practice

The soil microbial diversity influences the surrounding environmental conditions of soil bulk, including pH, temperature, and salinity, which subsequently manipulates plant growth and resistance to biotic and abiotic stresses [106]. Additionally, the soil microbiome also influences soil characteristics, and the use of cyanobacteria has become a far-reaching technology for stabilizing degraded soils [107]. The abundance of arbuscular mycorrhizal fungi (AMFs) is highly related to soil aggregation, while loss of AMFs in an ecosystem can have serious consequences for soil stabilization [25]. Application of AMFs significantly enhances soil quality through improving water retention capacity, facilitating nutrient supplementation, stimulating enzyme-dependent microbial activity, and promoting microbial yield [108]. In management practice, beneficial rhizobacteria induced by inhabiting crops (e.g., soybean) can be widely used to adjust the unbalanced soil microbiome and resolve continuous cropping obstacles or saline soils. These facts further indicate that crop rotation is instrumental in the regulation of the biodiversity of the root-associated microbiome. Indeed, soil-borne diseases, including fusarium wilt and root-knot nematodes, are the main factors limiting continuous cropping in fruit and vegetable facility cultivation. Recent studies show that phosphate metabolism and fungal-mediated adaptation promoting the growth and survival of plants are systematically integrated to increase the biodiversity of soil microorganisms [109,110,111]. In addition, soil microbiome applications for lignocellulosic biomass degradation are emerging as a potential strategy to overcome the requirements of biomass-based biofuel production technologies [112].

Given that the plant root- and crop residue-derived beneficial organic compounds serve as key regulators of soil microbiome biodiversity through establishing nutrient-rich microhabitats for rhizobacteria, enhancing the functional capacity of microbial communities offers a promising strategy to support horticultural plant physiology and organogenesis. The identification of bioactive metabolites in soil that can interact with cultivated plants and the isolation of beneficial microorganisms to absorb and digest these metabolites are of great significance for mitigating continuous cropping obstacles in horticultural management practice. Therefore, the effects and consequences of anthropogenic disturbance of soil microbiology are not only valuable in soil improvement and plant fitness regulation in agricultural systems, but also have a powerful role in green-energy production that relies on biomaterials. The integrity of soil health, including its physical structure, biochemical fertility, and biological activity, functions as the foundational promotion for both plant productivity and epigenetic reprogramming [113]. Dynamic soil properties may subsequently modulate plant epigenetic states, which need to be investigated in the future.

### 4.2. The Application of Epigenetic Regulation in Soil Microbiome for Horticultural Crops

**Figure 2:**
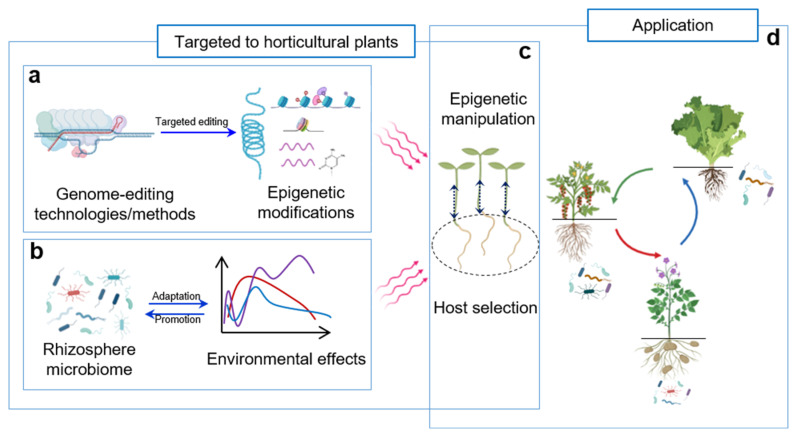
Illustration of the operational processes by which rhizosphere microbial communities and epigenetic modifications regulate the plasticity of horticultural plants. (**a**) Modification of specific epigenetic markers through genome-editing techniques. (**b**) Environmental fluctuations regulate microbial composition, and endogenous responses (e.g., metabolite variations or microbe-triggered immunity) enhance environmental adaptation. (**c**) Host selection, epigenetic regulation, and their synergistic communication drive accelerated evolution and extended phenotypic plasticity of horticultural crops. (**d**) The potential application of epigenetic modification-mediated plant root-associated microbiome in regulating many aspects of horticultural plant growth and development, such as fruit ripening, leaf senescence, and underground organ expansion. This schematic was partially generated using BioRender.com with some modifications.

The plant–rhizobacteria interaction is regulated by epigenetic modifications, as demonstrated by soil inoculated with *Bacillus megaterium* YC4 and *Trichoderma harzianum* T22, positively promoting plant growth in *Arabidopsis* and tomato [2], which exemplifies the practical implementation of plant–rhizobacteria interaction in horticultural crops. Due to the complexity of gene transcription regulation, the selection of effective plant growth-promoting bacteria and fungi targeted by epigenetic modifications is a long-term challenge in agricultural practice. The interaction between a host and root-associated microorganisms has been shown to promote the formation of crop products [114]. A new variety of vegetative stem crop named ‘Jiaobai’ was developed by the continuous infection with the endophytic fungus *Ustilago esculenta* [115]. It was found that *U. esculenta* invaded the underground roots in spring and gradually extended to newly germinated aboveground buds, eventually leading to the expansion of the fleshstem [116]. By combining these cases with models of epigenetic modifications that regulate plant phenotypes, coupled with the facts that environmental fluctuations influence microbial composition and endogenous responses (e.g., metabolite variations or microbe-triggered immunity) enhance environmental adaptation (Figure 2a,b), this phenomenon presents a promising approach to promote the production of underground edible organs in horticultural crops by improving rhizosphere microbial communities and their interaction with host crops under epigenetic guidance (Figure 2c,d). Specifically, the edible organs of tuber crops (e.g., potato and sweet potato) are produced underground and serve as non-grain food products that are important for global food security [117] (Figure 2d). The case of tomato roots recruiting beneficial rhizobacteria through inositol also suggests that epigenetic regulation mechanisms such as methylation are conserved across species [2]. Epigenetic modifications to the host and microbiome during adaptation to environmental conditions and artificial selection contribute to increasing the biodiversity of soils. For instance, histone variant H2A.Z plays an essential role in phosphate metabolism [118], which could contribute to bioresource integration between plant mineral nutrient absorption and soil microorganisms regulation [119]. Therefore, the rational application of candidate microorganisms and epigenetic markers generated by genome editing technology [120] to horticultural plants is a feasible direction for future management practices.

In addition, the epigenetic regulation of plant–microbial interactions is applicable to leafy horticultural crops. Communication between roots and leaves also participates in plant responses to adverse environments. The systematic root-to-stem signaling pathway enhanced defense responses to nematode attack in tomato plants [121]. Based on the mechanism of root–leaf signal transduction, reasonable control of beneficial rhizosphere microorganisms in practical management can not only induce systemic immunity to prevent diseases, but also effectively reduce pesticide application and enhance the safety of edible leaf vegetables. In fact, many studies have found that the location of rhizosphere microorganisms and the induced immune system are related to epigenetic mechanisms [2,16,87]. The use of a beneficial microbiome to improve crop health is a popular concept for future agricultural strategies [122]. A thorough elucidation of the mechanisms of epigenetic modifications on microbial communities and the organic integration of relevant theories into the above strategies will help realize the idea of coping with biological stress through mutual defense communications between plant organs [121].

## 5. Future Perspectives

Plants provide organic compounds to soil microbes, which selectively interact with plants. This reciprocal interaction forms an essential link in the material and energy cycles of the natural environment. Beneficial plant–microbiome interactions can induce systemic resistance to (a)biotic stresses and/or promote normal plant growth. In addition, the microbiome has the function to influence or improve host phenotypes and morphologies [123,124], making it a potential tool for sustainable crop production. On this basis, using epigenetic modification will support the regulation of favorable plant–microbiome interactions and thus accelerate host selection and horticultural crops production (Figure 2).

Extensive transcriptional reprogramming is a core strategy for plants and microorganisms in response to developmental and environmental cues, while epigenetic modifications dynamically regulate chromatin architecture to precisely activate or silence specific genes. On one hand, plant genetic and epigenetic coding information dynamically regulates the immune system and metabolites, thereby inducing the recombination of microbial communities and promoting the phenotypic plasticity of plants in fluctuating environments. In turn, changes in microbial communities can dramatically affect phenotypic remodeling and epigenome changes in plants.

As the world food crisis continues, epigenetic mechanism-mediated plant–microbial interactions are a promising way to improve crop quality and yield. With the increasing demands for high-quality and large quantities of crops, there is a need to gain favor among farmers for their short growing cycle and freedom from seasonal constraints. The significance of epigenetic modifications in regulating the soil biodiversity of the cropping system becomes more important because of the high frequency of vegetable planting, which can cause continuous cropping obstacles and the aggravation of pests and diseases. Given that high-quality reference genomes of root-associated microorganisms are fundamental for advancing both the metagenomic profiling and the mechanistic understanding of crop rhizosphere microbiomes [125], similar large-scale cultivation and metagenomic sequencing approaches could be applied to horticultural crops (e.g., tomato, strawberry, and cucumber) to construct comprehensive root microbial and viral genome collections, significantly expanding the current limited genomic resources for these economically important species. Such efforts would uncover conserved microbial functions and virus–host interactions unique to horticultural root ecosystems, enabling targeted microbiome engineering to improve stress resilience, nutrient uptake, and yield—key for advancing precision horticulture and sustainable greenhouse production systems. In addition, climate change acts as a key environmental stressor that simultaneously induces plant epigenetic modifications and significantly alters the composition and functionality of rhizosphere microbiomes [126]. Climate change acts not only as an environmental stressor for plants, altering their epigenetic characteristics, but also significantly regulates the composition and functionality of rhizosphere microbiomes. Climate change influences plant adaptability through epigenetic modifications and rhizosphere microbial regulation [126,127,128]. Therefore, manipulating the microbiome to induce and create breeding materials with epigenetic variations and resistance to potential continuous cropping barriers without altering the genome may be a practical direction for future crop production and agricultural soil management.

## Figures and Tables

**Table 1:** Plant epigenetic modification and the related microorganisms.

Plant Host	Interacted Microorganism (s)	Epigenetic Modification	References
Tomato (*S. lycopersicum* cv. ‘Crovarese’)	*Trichoderma harzianum* (beneficial)	Histone modifications and DNA methylation	Palma et al. 2019 [63]
*Arabidopsis thaliana*	*Oxalobacteraceae* spp.	Histone modifications and DNA methylation	Lv et al. 2022 [64]
*Arabidopsis thaliana*	*Streptomycetaceae* spp.	DNA methylation	Kaushal et al. 2021 [16]
*Arabidopsis thaliana*, *Solanum lycopersicum*	*Bacillus megaterium*	DNA demethylation	Vilchez et al. 2020 [2]
*Phytolacca americana*	*Bacillus* sp., *Arthrobacter* sp.	DNA methylation	Chen et al. 2022 [65]
*Oryza sativa*	sulfate-reducing bacteria	Histone acetylation	Chen et al. 2025 [66]

## Data Availability

No data are available in this study.
